# An ammonium iron(II) pyrophosphate, (NH_4_)_2_[Fe_3_(P_2_O_7_)_2_(H_2_O)_2_], with a layered structure

**DOI:** 10.1107/S1600536811052482

**Published:** 2011-12-10

**Authors:** Biao Liu, Xin Zhang, Lei Wen, Wei Sun, Ya-Xi Huang

**Affiliations:** a Department of Materials Science and Engineering, College of Materials, Xiamen University, Xiamen 361005, Fujian Province, People’s Republic of China

## Abstract

Diammonium diaquabis(phosphato)triferrate(II), (NH_4_)_2_[Fe_3_(P_2_O_7_)_2_(H_2_O)_2_], was synthesized under solvo­thermal conditions at 463 K. The crystal structure, isotypic to its Mn and Ni analogues, is built from iron pyrophosphate layers parallel to (100), which are linked by ammonium ions sitting in the inter­layer space *via* O—H⋯O and N—H⋯O hydrogen bonds. There are two crystallographic Fe sites in the crystal structure, one at a special position (2*a*, 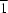), the other at a general position (4*e*, 1). The former Fe atom on the inversion centre is coordinated by six O atoms, forming an FeO_6_ octa­hedron, while the latter is coordinated by five phosphate O atoms and one water mol­ecule, forming an FeO_5_(H_2_O) octa­hedron. Each FeO_6_ octa­hedron shares *trans* edges with two FeO_5_(H_2_O) octa­hedra, forming a linear trimeric unit. These trimers share the lateral edges of FeO_5_(H_2_O) with other trimers, forming a zigzag chain running along [010]. The zigzag chains are further linked by P_2_O_7_ groups into a layered structure parallel to (100).

## Related literature

For background to this compound, see: Moore & Shen (1983 ▶); Lii *et al.* (1998 ▶); Alfonso *et al.* (2010 ▶); Mi *et al.* (2010 ▶). For background to the bond-valence method, see: Brown & Altermatt (1985 ▶). For related structures, see: Chippindale *et al.* (2003 ▶) for (NH_4_)_2_[Mn_3_(P_2_O_7_)_2_(H_2_O)_2_]; Lightfoot *et al.* (1990 ▶) for K_2_Co_3_(P_2_O_7_)_2_·2H_2_O; Liu *et al.* (2004 ▶) for Na(NH_4_)[Ni_3_(P_2_O_7_)_2_(H_2_O)_2_]; Wei *et al.* (2010 ▶) for (NH_4_)_2_[Ni_3_(P_2_O_7_)_2_(H_2_O)_2_].

## Experimental

### 

#### Crystal data

(NH_4_)_2_[Fe_3_(P_2_O_7_)_2_(H_2_O)_2_]
*M* *_r_* = 587.55
Monoclinic, 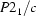

*a* = 9.4131 (17) Å
*b* = 8.1940 (15) Å
*c* = 9.3987 (17) Å
β = 99.651 (3)°
*V* = 714.7 (2) Å^3^
*Z* = 2
Mo *K*α radiation
μ = 3.55 mm^−1^
*T* = 173 K
0.08 × 0.06 × 0.06 mm

#### Data collection

Bruker Smart APEXI diffractometer equipped with CCD area-detector
Absorption correction: numerical (*SMART*; Bruker, 2001 ▶) *T*_min_ = 0.775, *T*_max_ = 0.808
4127 measured reflections
1652 independent reflections
1417 reflections with *I* > 2σ(*I*)
*R*_int_ = 0.035

#### Refinement

*R*[*F* ^2^ > 2σ(*F* ^2^)] = 0.040
*wR*(*F* ^2^) = 0.085
*S* = 1.05
1652 reflections
135 parameters
All H-atom parameters refined
Δρ_max_ = 0.55 e Å^−3^
Δρ_min_ = −0.55 e Å^−3^

### 

Data collection: *SMART* (Bruker, 2001 ▶); cell refinement: *SAINT* (Bruker, 2001 ▶); data reduction: *SAINT*; program(s) used to solve structure: *SHELXS97* (Sheldrick, 2008 ▶); program(s) used to refine structure: *SHELXL97* (Sheldrick, 2008 ▶); molecular graphics: *DIAMOND* (Brandenburg, 2005 ▶) and *ATOMS* (Dowty, 2004 ▶); software used to prepare material for publication: *SHELXL97* and *WinGX* (Farrugia, 1999 ▶).

## Supplementary Material

Crystal structure: contains datablock(s) I, global. DOI: 10.1107/S1600536811052482/fi2119sup1.cif

Structure factors: contains datablock(s) I. DOI: 10.1107/S1600536811052482/fi2119Isup2.hkl

Additional supplementary materials:  crystallographic information; 3D view; checkCIF report

## Figures and Tables

**Table:** 

Fe1—O1^i^	2.055 (3)
Fe1—O4^ii^	2.092 (3)
Fe1—O6^iii^	2.108 (3)
Fe1—O8	2.134 (3)
Fe1—O6^i^	2.185 (3)
Fe1—O5	2.261 (3)
Fe2—O5	2.127 (3)
Fe2—O4	2.135 (3)
Fe2—O3^iv^	2.201 (3)
P1—O3	1.514 (3)
P1—O6	1.517 (3)
P1—O4	1.523 (3)
P1—O2	1.620 (3)
P2—O7	1.510 (3)
P2—O1	1.516 (3)
P2—O5	1.518 (3)
P2—O2	1.631 (3)

**Table:** 

P1—O2—P2	128.83 (18)

Symmetry codes: (i) 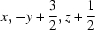; (ii) 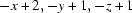; (iii) 
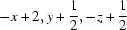
; (iv) 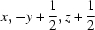.

**Table 2:** Hydrogen-bond geometry (Å, °)

*D*—H⋯*A*	*D*—H	H⋯*A*	*D*⋯*A*	*D*—H⋯*A*
O8—H1⋯O3^v^	0.89 (6)	1.87 (6)	2.734 (4)	162 (5)
O8—H2⋯O7^vi^	0.82 (6)	2.00 (6)	2.785 (4)	159 (5)
N1—H3⋯O1^vii^	0.91 (7)	1.86 (7)	2.717 (5)	156 (6)
N1—H4⋯O8^viii^	0.89 (6)	2.56 (7)	3.310 (6)	142 (5)
N1—H5⋯O7^ix^	0.96 (7)	1.99 (7)	2.859 (5)	150 (5)
N1—H6⋯O7	0.88 (7)	1.91 (7)	2.791 (5)	174 (6)

Symmetry codes: (v) 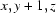; (vi) 
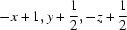
; (vii) 
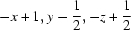
; (viii) 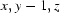; (ix) 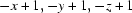.

**Fig. 1.**
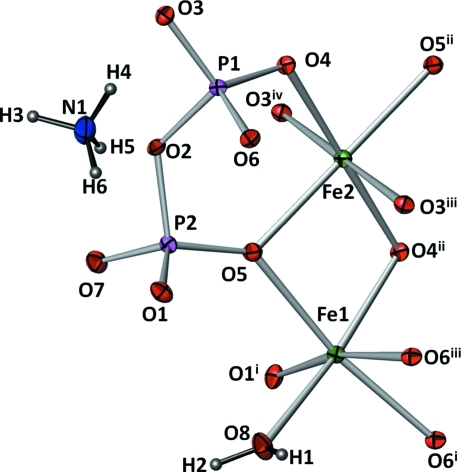
Structural unit of (NH4)2[FeII3(P2O7)2(H2O)2], showing the coordination environments of Fe and P atoms. Thermal ellipsoids at the 50% probability level. Green sphere: Fe atom, purple sphere: P atom, red sphere: O atom, blue sphere: N atom, dark grey sphere: H atom. Symmetry codes: (i) x, –y+3/2, z+1/2; (ii) –x+2, –y+1, –z+1; (iii) –x+2, y+1/2, –z+1/2; (iv) x, –y+1/2, z+1/2

**Fig. 2.**
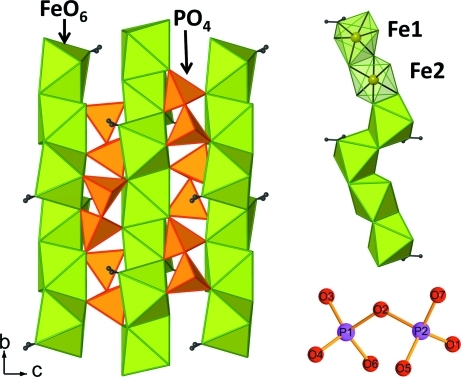
Polyhedral presentation of the iron pyrophosphate layer built from edge-sharing iron octhedral chains intra- and inter-connected by P2O7 groups. Green octahedron: FeO6, orange tetrahedron: PO4, green sphere: Fe atom, purple sphere: P atom, red sphere: O atom, dark grey sphere: H atom.

**Fig. 3.**
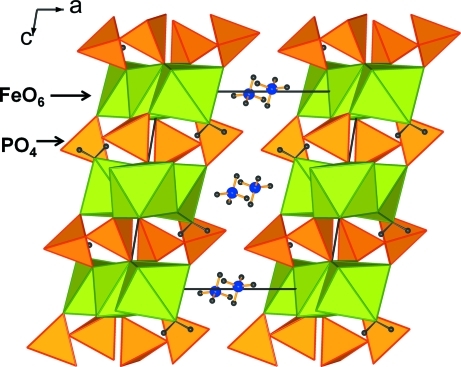
The crystal structure of (NH4)2[FeII3(P2O7)2(H2O)2] is built from zigzag chains of edge-sharing iron octahedra linked by P2O7 pyrophosphate groups to form layers parallel to (100), which are further linked by ammonium ions, sitting at the interlayer space, via the hydrogen bonds. Green octahedron: FeO6, orange tetrahedron: PO4, blue sphere: N atom, dark grey sphere: H atom.

## supplementary crystallographic information

### Comment

In the mineral kingdom, iron phosphates are one of the most important materials besides silicates and aluminates. The mineral cacoxenite is the most striking example in open-framework iron phosphate because of its gigantic cylindrical tunnels (diameter of 14.2 Å) which are only occupied by water molecules (Moore & Shen, 1983). Intrigued by the cacoxenite, a large amount of synthetic iron phosphates have been reported with 1-D, 2-D, and 3-D structures in last two decades (Lii *et al.*, 1998; Alfonso *et al.*, 2010; Mi *et al.*, 2010). Here we report on a new ammounium iron(II) pyrophosphate, (NH_4_)_2_[Fe^II^_3_(P_2_O_7_)_2_(H_2_O)_2_], with a layered structure.

The layered structure of the title compound is isotypic to (NH_4_)_2_[Mn_3_(P_2_O_7_)_2_(H_2_O)_2_] (Chippindale *et al.*, 2003), K_2_[Co_3_(P_2_O_7_)_2_(H_2_O)_2_] (Lightfoot *et al.*, 1990), (NH_4_)_2_[Ni_3_(P_2_O_7_)_2_(H_2_O)_2_] (Wei *et al.*, 2010), and Na(NH_4_)_2_[Ni_3_(P_2_O_7_)_2_(H_2_O)_2_] (Liu *et al.*, 2004). The asymmetric unit of the title compound (presented in Figure 1) shows that there are two crystallographically independent iron atoms with octahedral coordination and two distinct phosphorus atoms both with tetrahedral coordination. The two phosphate tetrahedra share the common O2-corner to form a P_2_O_7_ group. The Fe2 atom sits at the inversion center (0, 0.5, 0.5), while Fe1 atom at a general position. Fe2-octahedron shares its *trans*-edges to two Fe1-octahedra, while Fe1 shares *cis*-edges to a Fe1-octahedron and one Fe2-octahedron, resulting in zigzag chains running along [010] (Figure 2). The edge-sharing iron octahedral chains are intra- and inter-connected by P_2_O_7_ groups through common O-vertices to form flaty layers parallel to (100). The interlayer spaces are occupied by ammonium ions to compensate the negative charge of the iron pyrophosphate layers (Figure 3). Bond valence sum calculations suggest that both iron sites are in the 2+ oxidation state (2.05 for Fe1 and 1.95 for Fe2) (Brown & Altermatt, 1985) which are also consistent to the valence state of Mn^II^, Co^II^, and Ni^II^ in the related compounds (Chippindale *et al.*, 2003; Lightfoot *et al.*, 1990; Wei *et al.*, 2010; Liu *et al.*, 2004).

### Experimental

(NH_4_)_2_[Fe^II^_3_(P_2_O_7_)_2_(H_2_O)_2_] has been synthesized under solvothermal conditions. 0.112 g Fe powder and 0.345 g NH_4_H_2_PO_4_ were added to a solution of 3 mL pyridine and 2 mL 1,2-dihydroxypropane (with a molar ratio of Fe : P : N = 2 : 3 : 3). Then 1.25 mL H_3_PO_4_ (85%) was added to adjust the pH value to 6-7. The mixture was transferred to a 15 mL Teflon-lined stainless steel autoclave (filling degree of 40%) and heated at 463 K for 5 days. After that, the solution was slowly cooled to room temperature and washed with distilled water several times. Light yellow block-like crystals were obtained as a single phase which has been confirmed by powder X-ray diffraction.

### Refinement

The hydrogen atoms bonded to water (O8) and nitrogen (N1) were located from the difference Fourier maps and refined without applying any restraints on the bond length. The displacement parameter of the hydrogen atoms (H1, H2) coordinated to O8 were refined with the common U*_iso_* variables. The same treatment was applied to refine the displacement parameter of hydrogen atoms bonded to N1 (H3, H4, H5, H6).

### Crystal data

**Table:**

(NH_4_)_2_[Fe_3_(H_2_O)_2_(P_2_O_7_)_2_]	*F*(000) = 584
*M**_r_* = 587.55	*D*_x_ = 2.730 Mg m^−^^3^
Monoclinic, *P*2_1_/*c*	Mo *K*α radiation, λ = 0.71073 Å
Hall symbol: -P 2ybc	Cell parameters from 4127 reflections
*a* = 9.4131 (17) Å	θ = 2.2–28.3°
*b* = 8.1940 (15) Å	µ = 3.55 mm^−^^1^
*c* = 9.3987 (17) Å	*T* = 173 K
β = 99.651 (3)°	Block, light yellow
*V* = 714.7 (2) Å^3^	0.08 × 0.06 × 0.06 mm
*Z* = 2	

### Data collection

**Table:**

Bruker Smart APEXI diffractometer equipped with CCD area-detector	1652 independent reflections
Radiation source: fine-focus sealed tube	1417 reflections with *I* > 2σ(*I*)
graphite	*R*_int_ = 0.035
1265 images,φ=0, 90, 180°, and Δω=0.3°, χ= 54.74° scans	θ_max_ = 28.3°, θ_min_ = 2.2°
Absorption correction: numerical (*SMART*; Bruker, 2001)	*h* = −12→12
*T*_min_ = 0.775, *T*_max_ = 0.808	*k* = −5→10
4127 measured reflections	*l* = −12→12

### Refinement

**Table:**

Refinement on *F*^2^	Primary atom site location: structure-invariant direct methods
Least-squares matrix: full	Secondary atom site location: difference Fourier map
*R*[*F*^2^ > 2σ(*F*^2^)] = 0.040	Hydrogen site location: difference Fourier map
*wR*(*F*^2^) = 0.085	All H-atom parameters refined
*S* = 1.05	*w* = 1/[σ^2^(*F*_o_^2^) + (0.0331*P*)^2^ + 1.7041*P*] where *P* = (*F*_o_^2^ + 2*F*_c_^2^)/3
1652 reflections	(Δ/σ)_max_ < 0.001
135 parameters	Δρ_max_ = 0.55 e Å^−^^3^
0 restraints	Δρ_min_ = −0.55 e Å^−^^3^

### Special details

**Table:**

Geometry. All esds (except the esd in the dihedral angle between two l.s. planes) are estimated using the full covariance matrix. The cell esds are taken into account individually in the estimation of esds in distances, angles and torsion angles; correlations between esds in cell parameters are only used when they are defined by crystal symmetry. An approximate (isotropic) treatment of cell esds is used for estimating esds involving l.s. planes.
Refinement. Refinement of F^2^ against ALL reflections. The weighted R-factor wR and goodness of fit S are based on F^2^, conventional R-factors R are based on F, with F set to zero for negative F^2^. The threshold expression of F^2^ > 2sigma(F^2^) is used only for calculating R-factors(gt) etc. and is not relevant to the choice of reflections for refinement. R-factors based on F^2^ are statistically about twice as large as those based on F, and R- factors based on ALL data will be even larger.

### Fractional atomic coordinates and isotropic or equivalent isotropic displacement parameters (Å^2^)

**Table:**

	*x*	*y*	*z*	*U*_iso_*/*U*_eq_	
Fe1	0.86847 (6)	0.86015 (7)	0.50162 (6)	0.00855 (16)	
Fe2	1.0000	0.5000	0.5000	0.00833 (19)	
P1	0.90578 (11)	0.29865 (13)	0.20925 (10)	0.0077 (2)	
P2	0.70438 (11)	0.55532 (13)	0.26344 (11)	0.0085 (2)	
O1	0.7060 (3)	0.6456 (4)	0.1229 (3)	0.0126 (6)	
O2	0.7509 (3)	0.3679 (3)	0.2350 (3)	0.0092 (6)	
O3	0.8740 (3)	0.1331 (3)	0.1398 (3)	0.0105 (6)	
O4	0.9971 (3)	0.2951 (3)	0.3592 (3)	0.0102 (6)	
O5	0.8132 (3)	0.6192 (3)	0.3887 (3)	0.0099 (6)	
O6	0.9625 (3)	0.4199 (3)	0.1104 (3)	0.0099 (6)	
O7	0.5538 (3)	0.5385 (4)	0.2972 (3)	0.0126 (6)	
O8	0.7301 (3)	0.9906 (4)	0.3371 (3)	0.0168 (7)	
H1	0.777 (6)	1.016 (7)	0.265 (6)	0.033 (11)*	
H2	0.647 (6)	0.979 (7)	0.296 (6)	0.033 (11)*	
N1	0.5548 (5)	0.2761 (5)	0.4867 (5)	0.0203 (9)	
H3	0.480 (7)	0.206 (8)	0.461 (7)	0.050 (10)*	
H4	0.632 (7)	0.213 (8)	0.485 (7)	0.050 (10)*	
H5	0.550 (7)	0.323 (8)	0.579 (7)	0.050 (10)*	
H6	0.550 (7)	0.363 (8)	0.430 (7)	0.050 (10)*	

### Atomic displacement parameters (Å^2^)

**Table:**

	*U*^11^	*U*^22^	*U*^33^	*U*^12^	*U*^13^	*U*^23^
Fe1	0.0091 (3)	0.0087 (3)	0.0078 (3)	0.0007 (2)	0.0012 (2)	0.0006 (2)
Fe2	0.0095 (4)	0.0079 (4)	0.0074 (4)	0.0003 (3)	0.0008 (3)	−0.0001 (3)
P1	0.0082 (5)	0.0082 (5)	0.0065 (5)	0.0001 (4)	0.0010 (4)	−0.0001 (4)
P2	0.0084 (5)	0.0095 (5)	0.0075 (5)	0.0010 (4)	0.0005 (4)	0.0003 (4)
O1	0.0145 (14)	0.0132 (15)	0.0109 (14)	0.0029 (12)	0.0045 (11)	0.0007 (12)
O2	0.0085 (13)	0.0095 (14)	0.0099 (13)	−0.0015 (11)	0.0023 (11)	−0.0022 (11)
O3	0.0157 (14)	0.0086 (14)	0.0070 (13)	0.0002 (12)	0.0015 (11)	−0.0010 (11)
O4	0.0116 (14)	0.0118 (15)	0.0069 (13)	0.0019 (12)	0.0003 (11)	−0.0003 (11)
O5	0.0112 (14)	0.0085 (14)	0.0084 (13)	0.0018 (11)	−0.0032 (11)	−0.0022 (11)
O6	0.0141 (14)	0.0057 (14)	0.0108 (14)	0.0011 (11)	0.0048 (11)	0.0003 (11)
O7	0.0087 (13)	0.0170 (16)	0.0124 (14)	0.0010 (12)	0.0026 (11)	−0.0006 (12)
O8	0.0112 (15)	0.0241 (19)	0.0140 (15)	−0.0017 (14)	−0.0016 (12)	0.0092 (13)
N1	0.017 (2)	0.021 (2)	0.021 (2)	−0.0059 (17)	−0.0030 (17)	0.0012 (18)

### Geometric parameters (Å, °)

**Table:**

Fe1—O1^i^	2.055 (3)	P1—O6	1.517 (3)
Fe1—O4^ii^	2.092 (3)	P1—O4	1.523 (3)
Fe1—O6^iii^	2.108 (3)	P1—O2	1.620 (3)
Fe1—O8	2.134 (3)	P2—O7	1.510 (3)
Fe1—O6^i^	2.185 (3)	P2—O1	1.516 (3)
Fe1—O5	2.261 (3)	P2—O5	1.518 (3)
Fe2—O5	2.127 (3)	P2—O2	1.631 (3)
Fe2—O5^ii^	2.127 (3)	O8—H1	0.89 (6)
Fe2—O4	2.135 (3)	O8—H2	0.82 (6)
Fe2—O4^ii^	2.135 (3)	N1—H3	0.91 (7)
Fe2—O3^iv^	2.201 (3)	N1—H4	0.89 (6)
Fe2—O3^iii^	2.201 (3)	N1—H5	0.96 (7)
P1—O3	1.514 (3)	N1—H6	0.88 (7)
			
O1^i^—Fe1—O4^ii^	93.89 (11)	O6—P1—O4	112.16 (16)
O1^i^—Fe1—O6^iii^	167.57 (12)	O3—P1—O2	105.08 (15)
O4^ii^—Fe1—O6^iii^	91.46 (11)	O6—P1—O2	106.26 (16)
O1^i^—Fe1—O8	89.59 (12)	O4—P1—O2	104.54 (15)
O4^ii^—Fe1—O8	171.64 (12)	O7—P2—O1	111.99 (16)
O6^iii^—Fe1—O8	86.66 (12)	O7—P2—O5	113.84 (16)
O1^i^—Fe1—O6^i^	92.29 (11)	O1—P2—O5	113.78 (17)
O4^ii^—Fe1—O6^i^	93.09 (11)	O7—P2—O2	103.63 (16)
O6^iii^—Fe1—O6^i^	76.20 (11)	O1—P2—O2	105.91 (15)
O8—Fe1—O6^i^	94.37 (12)	O5—P2—O2	106.68 (15)
O1^i^—Fe1—O5	96.12 (11)	P2—O1—Fe1^v^	126.10 (17)
O4^ii^—Fe1—O5	80.21 (10)	P1—O2—P2	128.83 (18)
O6^iii^—Fe1—O5	95.84 (10)	P1—O3—Fe2^vi^	127.87 (17)
O8—Fe1—O5	91.87 (12)	P1—O4—Fe1^ii^	141.13 (18)
O6^i^—Fe1—O5	169.56 (10)	P1—O4—Fe2	120.34 (16)
O5—Fe2—O5^ii^	180.0	Fe1^ii^—O4—Fe2	98.46 (11)
O5—Fe2—O4	97.61 (10)	P2—O5—Fe2	128.17 (16)
O5^ii^—Fe2—O4	82.39 (10)	P2—O5—Fe1	137.51 (16)
O5—Fe2—O4^ii^	82.39 (10)	Fe2—O5—Fe1	93.67 (10)
O5^ii^—Fe2—O4^ii^	97.61 (10)	P1—O6—Fe1^vi^	121.76 (16)
O4—Fe2—O4^ii^	180.0	P1—O6—Fe1^v^	132.09 (16)
O5—Fe2—O3^iv^	92.14 (11)	Fe1^vi^—O6—Fe1^v^	103.80 (11)
O5^ii^—Fe2—O3^iv^	87.86 (10)	Fe1—O8—H1	110 (4)
O4—Fe2—O3^iv^	91.63 (10)	Fe1—O8—H2	134 (4)
O4^ii^—Fe2—O3^iv^	88.37 (10)	H1—O8—H2	103 (5)
O5—Fe2—O3^iii^	87.86 (10)	H3—N1—H4	103 (5)
O5^ii^—Fe2—O3^iii^	92.14 (11)	H3—N1—H5	110 (5)
O4—Fe2—O3^iii^	88.37 (10)	H4—N1—H5	114 (5)
O4^ii^—Fe2—O3^iii^	91.63 (10)	H3—N1—H6	113 (5)
O3^iv^—Fe2—O3^iii^	180.0	H4—N1—H6	114 (6)
O3—P1—O6	112.80 (16)	H5—N1—H6	103 (5)
O3—P1—O4	114.97 (16)		

Symmetry codes: (i) *x*, −*y*+3/2, *z*+1/2; (ii) −*x*+2, −*y*+1, −*z*+1; (iii) −*x*+2, *y*+1/2, −*z*+1/2; (iv) *x*, −*y*+1/2, *z*+1/2; (v) *x*, −*y*+3/2, *z*−1/2; (vi) −*x*+2, *y*−1/2, −*z*+1/2.

### Hydrogen-bond geometry (Å, °)

**Table:**

*D*—H···*A*	*D*—H	H···*A*	*D*···*A*	*D*—H···*A*
O8—H1···O3^vii^	0.89 (6)	1.87 (6)	2.734 (4)	162 (5)
O8—H2···O7^viii^	0.82 (6)	2.00 (6)	2.785 (4)	159 (5)
N1—H3···O1^ix^	0.91 (7)	1.86 (7)	2.717 (5)	156 (6)
N1—H4···O2^iv^	0.89 (6)	2.51 (6)	2.966 (5)	112 (5)
N1—H4···O8^x^	0.89 (6)	2.56 (7)	3.310 (6)	142 (5)
N1—H5···O7^xi^	0.96 (7)	1.99 (7)	2.859 (5)	150 (5)
N1—H6···O7	0.88 (7)	1.91 (7)	2.791 (5)	174 (6)

Symmetry codes: (vii) *x*, *y*+1, *z*; (viii) −*x*+1, *y*+1/2, −*z*+1/2; (ix) −*x*+1, *y*−1/2, −*z*+1/2; (iv) *x*, −*y*+1/2, *z*+1/2; (x) *x*, *y*−1, *z*; (xi) −*x*+1, −*y*+1, −*z*+1.
